# Adherence to the Mediterranean Diet Is Related to Healthy Habits, Learning Processes, and Academic Achievement in Adolescents: A Cross-Sectional Study

**DOI:** 10.3390/nu10111566

**Published:** 2018-10-23

**Authors:** Ramón Chacón-Cuberos, Félix Zurita-Ortega, Asunción Martínez-Martínez, Eva María Olmedo-Moreno, Manuel Castro-Sánchez

**Affiliations:** 1 Department of Didactics of Musical, Plastic and Corporal Expression, University of Granada, 18071 Granada, Spain; rchacon@ugr.es (R.C.-C.); felixzo@ugr.es (F.Z.-O.); 2 Department of Research Methods and Educational Diagnosis, University of Granada, 18071 Granada, Spain; asuncionmm@ugr.es (A.M.-M.); emolmedo@ugr.es (E.M.O.-M.)

**Keywords:** dietary patterns, Mediterranean diet, physical activity, motivation, learning strategies, adolescents

## Abstract

Background: Several studies have shown that following a healthy diet and practicing regular physical activity (PA) are related with multiple health benefits. However, the cognitive and academic implications of these behaviors within adolescents requires further study. Material and Methods: A cross-sectional study was conducted with a simple of 1059 adolescents from Spain. The main instruments employed were the Adherence to Mediterranean Diet Test (KIDMED), the Physical Activity Questionnaire for Adolescents (PAQ-A) and the Motivation and Learning Strategies Short Form (MSLQ-SF). Results: Practicing PA for more than three hours per week was related to better dietary habits (*p* < 0.001) such as increased consumption of vegetables (0.75 ± 0.43 vs. 0.62 ± 0.48), fish (0.67 ± 0.47 vs. 0.58 ± 0.49), cereals (0.85 ± 0.35 vs. 0.77 ± 0.41) and nuts (0.44 ± 0.49 vs. 0.35 ± 0.47). High adherence to a Mediterranean diet (MD) was positively related to elaboration strategies (*r* = 0.116), organizational strategies (*r* = 0.109), critical thinking (*r* = 0.116), self-regulation (*r* = 0.159), time and study habits (*r* = 0.160), self-regulation of effort (*r* = 0.118), and intrinsically orientated goals (*r* = 0.090) (*p* < 0.01 for all variables). Practicing PA every week was also related to improvements in several of the measured variables and in addition was related to lower levels of anxiety within the academic environment (*r* = −0.070; *p* < 0.05). Conclusions: Given the benefits of eating habits and the practice of PA in the cognitive processes involved in adolescent learning, intervention programs within the educational context are recommended to improve healthy habits.

## 1. Introduction

In recent decades, several studies have highlighted the importance of improving aspects of the teaching-learning process at different educational stages in order to encourage better learning outcomes in students [1,2]. Intrinsic motivation of students has been identified as an important factor in maintaining student interest in learning materials provided within the classroom, and improving capacity for self-regulation of academic behaviours [2,3,4]. Likewise, the promotion of organizational strategies and the development of critical thinking will reduce the level of academic anxiety experienced during evaluation periods and the completion of academic work [5]. It is therefore necessary to develop educational resources directed towards these aforementioned characteristics and towards other factors of cognitive performance and emotional well-being, such as physical-healthy habits [6,7].

Previous research (e.g., Bhushan et al. [8] and Esposito et al. [9]) identify a number of physical and cognitive benefits of following healthy habits; these include improving body composition, emotional well-being and self-concept, and reducing the risk of cardiovascular disease, stress, and anxiety [10,11,12]. The Mediterranean diet (MD) constitutes a balanced and healthy dietary model, which has been linked to the benefits described [11,13]. It is characterized by a high consumption of vegetables, legumes, cereals, fish, fruits, and nuts, as well as a moderate consumption of eggs, milk, and meat [14]. Moreover, MD recommendations encourage a reduced consumption of processed and sweet foods, alcohol, and tobacco. This has also been linked to a better state of general health [15]. Several studies have identified the importance of promoting this diet at early life stages in order to encourage positive physical, cognitive, and academic development in children and young people [16,17].

The practice of physical activity (PA) and sport has become an essential component of a healthy lifestyle alongside healthy dietary patterns [18]. Physical activity is defined as any bodily movement produced by skeletal muscles that requires energy expenditure [19]. PA improves cardio-respiratory fitness, insulin sensitivity, muscle tone, and cholesterol levels [20]. In addition, an active lifestyle can help reduce states of depression and anxiety [21], increase self-esteem [22], and improve academic performance [23]. The World Health Organization (WHO) [24] recommends a minimum of 60 minutes a day of moderate PA for children aged between 5 and 17 years, and a minimum of 150 minutes per week for individuals aged 18 years and above. It is therefore important to promote PA within educational and health contexts, in order to encourage an improved health status, better well-being, and greater academic success [25,26].

Associations between diet, PA engagement, and cognitive and academic performance have been identified. For example, Nyaradi et al. [27] reported that adolescents from western areas of Australia typically consumed a low-quality diet and obtained poor academic outcomes. It is possible that these individuals were experiencing cognitive dysfunction related to metabolic syndrome which was linked to a greater consumption of take-away food, soft drinks, red meat and saturated fats [15,27]. Further, a systematic review conducted by Hardman et al. [28] also revealed high adherence to a MD to be associated with greater cognitive performance. Martínez-Lapiscina et al. [29] delivered an intervention program based on MD, nuts, and extra virgin olive oil and found improvements to attention capacity, memory, abstract thinking, spatial vision and calculation capacity. The improved cognitive abilities may have been due to greater intakes of vitamins, minerals and natural antioxidants.

Another aspect that should be studied in relation to the improvement of cognitive performance through diet is the intake of foods rich in polyphenol. Godos et al. [30] reveal how the MD is closely related to the intake of this component, which is present in fruits, vegetables or other foods typical of the Mediterranean area such as tea. In this sense, it has been demonstrated how the intake of polyphenol helps to reduce depressive symptoms and improve cognition thanks to its anti-inflammatory and antioxidant properties, which are beneficial for a normal development of learning processes [30,31]. Thus, considering the relatively low levels of adherence to MD that exist in children and adolescents in Southern European countries, it is shown the need to promote this dietary model in order to improve their health status and cognitive processes linked to academic performance [32].

Similarly, Donelly et al. [17] reported positive effects of PA engagement on cognition, as well as on learning and academic performance. Specifically, this study also highlighted positive effects of practicing aerobic exercise on brain structures and their functions improving attention and concentration capacity and enabling the achievement of higher academic performance. Similarly, Erickson et al. [33] identified PA engagement to be associated with greater cerebral blood supply, a greater volume in the frontal cortex and the hippocampus, and lower losses to cognitive capacity in adulthood. Finally, a systematic review by Singh et al. [34] demonstrates positive associations between following an active lifestyle and successful school performance. The importance of developing interventions targeting positive motivational aspects is, therefore, clear.

The present study had two main objectives:

- To establish the relationship between different dietary patterns and the practice of PA in a sample of adolescents.
- To determine the associations between the level of adherence to the MD, practice of PA, motivation and learning strategies in adolescents.

## 2. Materials and Methods

### 2.1. Subjects and Design

**Figure 1:**
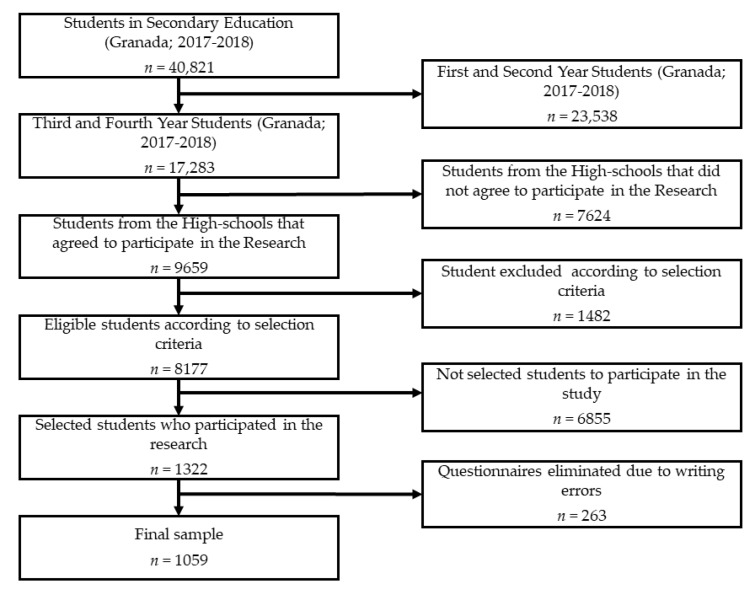
Study sample.

This research presents a non-experimental, descriptive and cross-sectional design. The sample consisted of 1059 adolescents from the province of Granada (Spain). Participants were aged between 14 and 16 years old ($\bar{x}$ = 15.23, SD = 1.08), 49.4% (*n* = 527) male and 50.6% (*n* = 532) female. Participating centres came from both public and private sectors. There was a total of 40,821 high school students from the province of Granada in the academic year 2017–2018. A total of 17,283 students who studied in the third and fourth years of this educational stage were considered in the research. Finally, a total of 1322 adolescents were selected by convenience, considering the educational centres that accepted to participate in this study and the selection criteria ((1) regularly attend to high school; (2) do not repeat course; (3) do not suffer from important pathologies). The final sample consisted of 1059 subjects. A total of 263 questionnaires had to be eliminated because they were completed incorrectly (Figure 1).

### 2.2. Instruments

Test of Adherence to Mediterranean Diet (KIDMED) [35]. This questionnaire is composed of 16 dichotomous items related to Mediterranean diet components offering a yes-no response; e.g., “You eat fresh or cooked vegetables every day”. Four of these items were negatively framed (−1) and twelve were positively framed (+1). Potential overall scores ranged from −4 to +12. The KIDMED has demonstrate internal consistency of α = 0.86.

Physical Activity Questionnaire for Adolescents (PAQ-A). The PAQ-A has been validated by Kowalski et al. [36] and translated into Spanish by Martínez-Gómez et al. [37]. Respondents self-report their engagement in PA as well as the type of PA engaged in during the prior seven days. The scale provides 10 items scored on a five-point Likert scale; e.g., “In the last seven days what did you usually do at lunchtime (before and after eating)”. All items are summed to provide a total score. The scale has demonstrated adequate reliability with a Cronbach’s alpha of α = 0.89.

Motivation and Learning Strategies Questionnaire-Short Form (MLSQ-SF). The MLSQ-SF has been validated by Pintrich et al. [38] and adapted to a short version of 40 items by Sabogal et al [39]. It consists of 40 items which are scored on a 5-points Likert scale (1 = Never and 5 = Always); e.g., “I strive academically even if I do not like what I do”. The items are grouped into 8 dimensions: value of task, anxiety, elaboration strategies, organizational strategies, critical thinking, self-regulation, time and study habits, self-regulation of effort, and intrinsic orientated goals. This instrument develops internal consistency of α = 0.94.

### 2.3. Procedure

Ethic approval was granted by the Ethics Committee of the University of Granada (reference: 641/CEIH/2018). The Department of Didactics of Musical, Plastic and Corporal Expression of the University of Granada sent information packs to invite education centres to participate. After the centres expressed interest to participate, information packs with full study details were provided to legal guardians of potential participants to provide informed consent.

Data was collected during school hours before the start of normal lessons. Participants were instructed on how to complete the questionnaires and a research assistant was on hand to provide guidance and resolve queries. No incentives were provided to participants. We have to point out that this research study has followed the ethical principles for research established by the Declaration of Helsinki. The participants’ right to confidentiality has also been respected. 

### 2.4. Data Analysis

The software IBM SPSS^®^ 22.0 (IBM Corp, Armonk, NY, USA) was used for statistical analysis. Frequencies and medians were used to describe the included variables. Associations between variables were analyzed using *t*-tests for independent samples, ANOVA and bivariate Pearson correlations. Normality of the data was analyzed using the Kolmogorov-Smirnov test and the Lillieforts’ correction. Levene’s test was employed to check homoscedasticity. Cronbach’s alpha coefficient was used to analyze internal reliability of all included instruments. The reliability index was established at 95.5%. The significance level was set at 0.05 [40].

## 3. Results

Table 1 shows the characteristics of the sample under study. It was obtained a gender representation of 49.4% (*n* = 527) for males and 50.6% (*n* = 532), while the distribution of the sample according to the type of centre was 63.8% (*n* = 675) for public high-schools and a 36.2% (*n* = 384) for private centres. In addition, a 20.7% (*n* = 219) of adolescents lived in rural areas and a 79.3% (*n* = 840) lived in urban areas. It was observed that 51.4% (*n* = 544) of the subjects were physically active, while 48.6% (*n* = 515) performed less than three hours per week of PA. Finally, the level of adherence to MD showed a 0.6% (*n* = 6) of respondents with a low adherence, a 22.7% (*n* = 240) with a medium adherence and a 76.8% (*n* = 813) with high adherence to MD.

Table 2 presents the relationships between the different dietary habits related to MD and the PA engagement of more than three hours per week. Adolescents who reported engaging in PA also reported higher consumption of cereals for breakfast (0.85 ± 0.35 vs. 0.77 ± 0.41, *p* < 0.001), greater probability of consuming a second fruit every day (0.40 ± 0.49 vs. 0.22 ± 0.41, *p* < 0.001), eating more fresh or cooked vegetables regularly (0.75 ± 0.43 vs. 0.62 ± 0.48, *p* < 0.001), more regular fish consumption (0.67 ± 0.47 vs. 0.58 ± 0.49, *p* < 0.001) and more regular consumption of nuts (0.44 ± 0.49 vs. 0.35 ± 0.47, *p* < 0.001). Moreover, PA engagement was associated with a lower score for the consumption of commercially baked goods or pastries (0.18 ± 0.38 vs. 0.27 ± 0.44; *p* < 0.001) as well as a higher score for the consumption of a second dairy product each day (0.58 ± 0.49 vs. 0.51 ± 0.50; *p* < 0.05). On the other hand, adolescents who engaged in PA also reported higher intake of fruit or fruit juice every day (0.30 ± 0.45 vs. 0.49 ± 0.50, *p* < 0.001), consumption of legumes (0.12 ± 0.33 vs. 0.21 ± 0.40, *p* < 0.001) and a higher consumption of sweets (0.79 ± 0.40 vs. 0.63 ± 0.48, *p* < 0.001). Finally, the overall score for adherence to a MD was higher for adolescents who were physically active (9.34 ± 1.90 vs. 8.67 ± 2.04). 

The relationships between adherence to MD and the different dimensions of motivation and learning strategies in adolescents are shown in Table 3. Adolescents reporting high MD adherence also reported significantly higher scores for the following variables than adolescents reporting low MD adherence: Elaboration strategies (4.10 ± 0.90 vs. 3.53 ± 1.28, *p* < 0.001), organizational strategies (4.08 ± 0.93 vs. 4.00 ± 1.54, *p* < 0.001), critical thinking (3.75 ± 0.98 vs. 2.78 ± 0.50, *p* < 0.001), self-regulation (3.78 ± 0.88 vs. 3.73 ± 1.54, *p* < 0.001), time and study habits (3.91 ± 0.87 vs. 3.72 ± 1.33, *p* < 0.001), self-regulation of effort (4.16 ± 0.82 vs. 3.61 ± 1.35, *p* < 0.001), and intrinsically orientated goals (4.24 ± 0.87 vs. 3.58 ± 1.28, *p* < 0.001).

Table 4 shows the associations between the different dimensions of motivation and learning strategies in adolescents and engagement in more than three hours a week of physical activity. Adolescents who engaged in PA also reported higher scores for critical thinking (3.75 ± 0.95 vs. 3.60 ± 1.02, *p* < 0.05), self-regulation (3.76 ± 0.87 vs. 3.61 ± 0.96, *p* < 0.05), time and study habits (3.89 ± 0.88 vs. 3.76 ± 0.94, *p* < 0.05), and intrinsically oriented goals (4.23 ± 0.84 vs. 4.12 ± 0.97, *p* < 0.05) and lower scores for anxiety (3.27 ± 1.08 vs. 3.42 ± 1.07; *p* < 0.05).

The associations between each dimension of motivation and learning strategies, adherence to a MD and engagement in physical activity are shown in Table 5. Adherence to a MD was positively related with elaboration strategies (*r* = 0.116; *p* < 0.01), organizational strategies (*r* = 0.109; *p* < 0.01), critical thinking (*r* = 0.116; *p* < 0.01), self-regulation (*r* = 0.159; *p* < 0.01), time and study habits (*r* = 0.160; *p* < 0.01), self-regulation of effort (*r* = 0.118; *p* < 0.01), intrinsically oriented goals (*r* = 0.1090; *p* < 0.01) and engagement in PA (*r* = 0.167; *p* < 0.01). In addition, engagement in PA was negatively associated with anxiety (*r* = −0.070; *p* < 0.05) and positively associated with critical thinking (*r* = 0.075; *p* < 0.05), self-regulation (*r* = 0.077; *p* < 0.05), and time and study habits (*r* = 0.074; *p* < 0.05). Further, anxiety was positively related to all dimensions of the motivation and learning strategies questionnaire while the value of task dimension was positively related with all other dimensions. Finally, all the other dimensions of this instrument were positively related.

## 4. Discussion

Several studies have shown the potential effects of a healthy diet and exercise on academic performance, highlighting the potential role of these behaviours for motivation and learning strategies. The main objective of the present research was to identify the existing relationships between dietary habits and engagement with PA in adolescents. A further objective was to analyse the associations between these healthy habits and different elements of motivation and learning, such as intrinsic motivation, anxiety, critical thinking, self-regulation and study habits. Previous studies have also called for further research in this area. e.g., Martínez-Lapiscina et al. [29], Erickson et al. [30], Santomauro et al. [41], Godoy et al. [42] and Chacón et al. [43].

Engagement in more than three hours a week of PA was related to a higher quality diet, characterised by a higher intake of cereals, fruit, dairy products, vegetables, fish and nuts. Similarly, the most highly active adolescents also reported consuming fewer processed products, although they also reported a higher consumption of sweets. Indeed, engagement in daily physical activity was associated with higher levels of overall adherence to MD. Research conducted by Kelishadi et al. [44] shows that the higher energy expenditure of active young people necessitates higher intakes of essential nutrients, which requires a higher consumption of carbohydrates, vitamins and quality proteins. This at least partly explains the positive relationship between MD and PA. Furthermore, family influences and contextual factors such as the school environment and the influence of peer groups often result in the clustering of behavioural habits meaning s that adolescents who follow a more active lifestyle tend to develop other healthy habits such as consuming a healthy diet [45,46,47].

It was observed that adolescents who reported a greater adherence to a MD also reported higher scores for elaboration and organization strategies, critical thinking and study habits. Following a healthier diet was also related to a greater capacity for effort, self-regulation and setting intrinsically oriented goals. Indeed, adequate nutrition has been found to positively influence cognitive capacity [48]. It has been demonstrated that higher adherence to Mediterranean diet is associated with higher intake of dietary polyphenols, which in turn are inversely associated with depression and cognitive decline. Higher polyphenol intake decreases systematic inflammation and the level of oxidative stress, therefore, possibly improving cognitive function [30]. In addition, the intake of phospholipids is essential for maintaining electrical potential of the membranes of neuronal cells and enabling memory formation. Moreover, the intake of fatty acids, such as omega-3, a prevalent acid in the MD, reduces the risk of cardiovascular disease, acting as a protective factor against cognitive deterioration and improving cerebral blood flow [48,49,50].

Another explanation of the benefits of the MD regarding learning strategies and cognitive performance lies in the importance of avoiding prolonged fasting, something proposed in the MD model [14]. Indeed, a typical MD recommends consuming five daily meals with an even balance of macronutrients in order to maintain blood glucose levels and improve attention and memory capacity [51]. Similarly, it is equally important to reduce the consumption of refined sugars in order to decrease postprandial memory, which has the potential to impede study habits and effort capacity [52]. It has also been shown that a healthy diet relates to higher levels of perceived well-being, which can help to encourage self-determined behaviors towards academic work, reduce levels of anxiety, and improve the self-regulation capacity in situations of stress related to the school environment [10,28,53].

There was a positive relationship between PA, motivation and learning strategies. Specifically, it was shown that engaging in an active lifestyle decreased academic anxiety and improved self-regulation. In addition, results revealed that adolescents who engaged in more PA spent more time studying, reported a higher critical thinking score and were more likely to set intrinsically oriented goals. These findings can have partially explained by research conducted previously. Stubbs et al. [21] and Rebar et al. [54] have discussed the release of endorphins and the increase in self-confidence following exercise which helps to free the mind of stressors and control depression and anxiety states. Likewise, engaging in PA hedonistically is associated with positive perceptions of personal well-being and feelings of self-determination. Self-regulation and intrinsic motivation towards PA should, therefore, be encouraged [25,55]. In combination with the aforementioned physiological responses to physical activity, these factors explain the greater longevity of participation in research studies of physically active adolescents [17,33,56].

A direct relationship was revealed between intrinsically oriented goals, learning strategies, critical thinking, study habits and self-regulation. Moreover, the task value dimension and anxiety were inversely related to the other measured variables. This finding can be explained by the negative state generated by stressors experienced in the academic context which diminish the capacity for attention and self-regulation of young people. In this context young people may believe themselves to be unable to overcome challenges, diminishing their motivation to maintain focus on educational materials [57,58]. It is, therefore, essential to promote self-determined motivations which improve the capacity for self-regulation and attention, enabling better cognitive and academic performance. In this regard, physical activity and healthy dietary patterns are elements which favour the avoidance of anxiety and the achievement of academic success.

The present research had the following limitations. Firstly, the study employed a descriptive and cross-sectional design which provides useful information on this novel topic but precludes casual conclusions from being made. Secondly, the sampling strategy used, followed recommendations of Merino-Marban et al. [40]. Nevertheless, it may have been more interesting to have performed a stratified random sampling procedure using the various areas of the province of Granada as the unit of randomisation. Another limitation lies in the variables employed, because although these are valid to fulfil the objectives of the study, it would have been interesting to include others of great relevance such as the body mass index or the total energy intake/day. This would allow to deepen more in the relationship between the motivations in the learning process, the alimentary habits and the state of health. Finally, whilst significant relationships were elaborated for the MD, PA, and dimensions of the MLSQ-SF, the strength of correlations were generally low. With these limitations taken into consideration, the following suggestions can be made for future research. The present study should be replicated and expanded within other samples and examining further variables, such as academic performance based on school grades. In addition, it would be interesting to develop an intervention program in order to verify the combined effect of MD and PA on the cognition and academic performance of adolescents.

## 5. Conclusions

The main conclusions from the present research highlight the importance of better dietary patterns and high adherence to a MD, particularly through a higher consumption of fruits, vegetables, fish, cereals and nuts. Moreover, a number of dimensions of motivation and learning strategies were positively associated with these healthy behaviors. Adolescents who followed a high-quality diet reported higher scores for organizational strategies, self-regulation, critical thinking, effort, study habits and intrinsically oriented goals, in addition to lower anxiety linked to academic contexts. The importance of promoting healthy dietary and physical activity habits is emphasised given identified physical, cognitive and academic benefits. Intervention programs targeted these behaviours within older children and adolescents should be a key priority for health and productivity.

## Figures and Tables

**Table 1:** Characteristic of the sample.

**Gender**	
Male	49.4% (*n* = 527)
Female	50.6% (*n* = 532)
**Type of school**	
Public	63.8% (*n* = 675)
Private	36.2% (*n* = 384)
**Region**	
Rural	20.7% (*n* = 219)
Urban	79.3% (*n* = 840)
**Level of PA (+ 3 h/Week)**	
Yes	48.6% (*n* = 515)
No	51.4% (*n* = 544)
**Adherence to MD**	
Low adherence	0.6% (*n* = 6)
Medium adherence	22.7% (*n* = 240)
High adherence	76.8 (*n* = 813)

Note 1: Level of PA (+ 3 h/week), More than 3 h/week of Physical Activity.

**Table 2:** Dietary patterns for adherence to MD according to PA.

	+ 3 h/Week PA	$\bar{x}$	SD	Standard Error	*t*-Test (Sig.)
I1. To have breakfast	Yes	0.91	0.28	0.013	0.094
	No	0.88	0.32	0.014	
I2. To have a dairy product for breakfast (yoghurt, milk, etc.)	Yes	0.77	0.42	0.019	0.164
	No	0.73	0.44	0.019	
I3. To have cereals or grains (bread, etc.) for breakfast	Yes	0.85	0.35	0..016	***
	No	0.77	0.41	0.018	
I4. To eat commercially baked goods or pastries for breakfast	Yes	0.18	0.38	0.017	***
	No	0.27	0.44	0.019	
I5. To eat a fruit or fruit juice every day	Yes	0.30	0.45	0.020	***
	No	0.49	0.50	0.021	
I6. To have a second fruit every day	Yes	0.40	0.49	0.022	***
	No	0.22	0.41	0.018	
I7. To have a second dairy product in the same day	Yes	0.58	0.49	0.022	*
	No	0.51	0.50	0.021	
I8. To eat fresh or cooked vegetables regularly once a day	Yes	0.75	0.43	0.019	***
	No	0.62	0.48	0.021	
I9. To consume fresh or cooked vegetables more than once a day	Yes	0.46	0.49	0.022	***
	No	0.33	0.47	0.020	
I10. Regular fish consumption (at least 2–3/week)	Yes	0.67	0.47	0.021	***
	No	0.58	0.49	0.021	
I11. To go >1/ week to a fast food restaurant	Yes	0.57	0.49	0.022	0.221
	No	0.53	0.50	0.021	
I12. To consume nuts regularly (at least 2–3/week)	Yes	0.44	0.49	0.022	***
	No	0.35	0.47	0.020	
I13. To eat legumes regularly (at least 2–3/week)	Yes	0.12	0.33	0.015	***
	No	0.21	0.40	0.017	
I14. To eat pasta or rice almost every day (5 or more per week)	Yes	0.57	0.49	0.022	0.786
	No	0.58	0.49	0.021	
I15. To have sweets and candy several times every day	Yes	0.79	0.40	0.018	***
	No	0.63	0.48	0.021	
I16. To use olive oil at home	Yes	0.99	0.10	0.005	0.924
	No	0.99	0.10	0.004	
Global score for adherence to MD	Yes	9.34	1.90	0.084	***
	No	8.67	2.04	0.087	

Note 1: * Statistically significant differences at level *p* < 0.05; ** Statistically significant differences at level *p* < 0.01; *** Statistically significant differences at level *p* < 0.001. Note 2: + 3 h/week PA, More than 3 h/week of Physical Activity.

**Table 3:** Relationships between adherence to MD, motivation and learning strategies.

		$\bar{x}$	SD	Standard Error	Confidence Interval (95%)		Sig.
					Lower Limit	Upper Limit	
VTA	L-A	3.17	1.37	0.563	1.72	4.61	0.080
	M-A	2.41	1.03	0.066	2.28	2.54	
	H-A	2.34	0.97	0.034	2.27	2.40	
ANS	L-A	2.58	1.25	0.511	1.27	3.90	0.208
	M-A	3.37	1.09	0.070	3.23	3.51	
	H-A	3.34	1.07	0.038	3.27	3.41	
ELS	L-A	3.53	1.28	0.523	2.19	4.88	***
	M-A	3.78	0.98	0.064	3.65	3.90	
	H-A	4.10	0.90	0.032	4.04	4.16	
ORS	L-A	4.00	1.54	0.632	2.37	5.63	***
	M-A	3.67	1.14	0.074	3.52	3.81	
	H-A	4.08	0.96	0.034	4.01	4.15	
CRT	L-A	2.78	0.50	0.205	2.25	3.30	***
	M-A	3.42	1.00	0.065	3.29	3.55	
	H-A	3.75	0.98	0.034	3.68	3.82	
SFR	L-A	3.73	1.54	0.630	2.21	5.45	***
	M-A	3.36	0.96	0.062	3.24	3.48	
	H-A	3.78	0.88	0.031	3.71	3.84	
TSH	L-A	3.72	1.33	0.545	2.32	5.12	***
	M-A	3.55	0.99	0.064	3.42	3.67	
	H-A	3.91	0.87	0.031	3.85	3.97	
SRE	L-A	3.61	1.35	0.554	2.19	5.03	***
	M-A	3.83	0.98	0.063	3.71	3.95	
	H-A	4.16	0.82	0.029	4.10	4.21	
GIO	L-A	3.58	1.28	0.523	2.24	4.93	***
	M-A	3.94	1.00	0.065	3.81	4.07	
	H-A	4.24	0.87	0.031	4.18	4.30	

Note 1: L-A: low adherence to MD; M-A: medium adherence to MD; H-A: high adherence to MD. Note 2: *** Statistically significant differences at level *p* < 0.001. Note 3: MD: Mediterranean diet; VTA: value of task; ANS: anxiety; ELS: elaboration strategies; OS: organizational strategies; CRT: critical thinking; SFR: self-regulation; TSH: time and study habits; SRE: self-regulation of effort; GIO: goals of intrinsic orientation.

**Table 4:** Relationships between adherence to MD, motivation and learning strategies.

	+ 3 h/Week PA	$\bar{x}$	DT	Standard Error	*t*-Test (Sig.)
VTA	Yes	2.35	0.98	0.043	0.806
	No	2.37	1.00	0.043	
ANS	Yes	3.27	1.08	0.048	0.023 *
	No	3.42	1.07	0.046	
ELS	Yes	4.07	0.89	0.039	0.111
	No	3.98	0.97	0.042	
ORS	Yes	4.03	0.96	0.042	0.191
	No	3.95	1.08	0.046	
CRT	Yes	3.75	0.95	0.042	0.014 *
	No	3.60	1.02	0.044	
SFR	Yes	3.76	0.87	0.038	0.012 *
	No	3.61	0.96	0.042	
TSH	Yes	3.89	0.88	0.039	0.015 *
	No	3.76	0.94	0.041	
SRE	Yes	4.12	0.82	0.036	0.115
	No	4.04	0.91	0.039	
GIO	Yes	4.23	0.84	0.037	0.048 *
	No	4.12	0.97	0.042	

Note 1: * Statistically significant S differences at level *p* < 0.05. Note 2: MD: Mediterranean diet; VTA: value of task; ANS: anxiety; ELS: elaboration strategies; OS: organizational strategies; CRT: critical thinking; SFR: self-regulation; TSH: time and study habits; SRE: self-regulation of effort; GIO: goals of intrinsic orientation. Note 3: + 3 h/week PA, More than 3 h/week of physical activity.

**Table 5:** Bivariate correlations between MD, PA, motivation and learning strategies.

	VTA	ANS	ELS	ORS	CRT	SFR	TSH	SRE	GIO	+ 3 h/Week PA
MD	−0.046	−0.047	0.116 **	0.109 **	0.116 **	0.159 **	0.160 **	0.118 **	0.090 **	0.167 **
VTA		0.281 **	−0.070 *	−0.028	0.065 *	0.015	−0.118 **	−0.128 **	0.006	−0.008
ANS			0.265 **	0.269 **	0.241 **	0.221 **	0.223 **	0.279 **	0.261 **	−0.070 *
ELS				0.721 **	0.673 **	0.738 **	0.693 **	0.755 **	0.654 **	0.049
ORS					0.618 **	0.639 **	0.695 **	0.661 **	0.574 **	0.040
CRT						0.664 **	0.633 **	0.609 **	0.560 **	0.075 *
SFR							0.710 **	0.717 **	0.673 **	0.077 *
TSH								0.726 **	0.593 **	0.074 *
SRE									0.645 **	0.048
GIO										0.059

Note 1: * Statistically significant differences at level *p* < 0.05; ** Statistically significant differences at level *p* < 0.01. Note 2: MD: Mediterranean diet; VTA: value of task; ANS: anxiety; ELS: elaboration strategies; ORS: organizational strategies; CRT: critical thinking; SFR: self-regulation; TSH: time and study habits; SRE: self-regulation of effort; GIO: Intrinsically oriented goals. Note 3: + 3 h/week PA, More than 3 h/week of physical activity.
